# Haploinsufficiency of the Parkinson’s disease gene synaptojanin1 is associated with abnormal responses to psychomotor stimulants and mesolimbic dopamine signaling

**DOI:** 10.3389/fnbeh.2024.1359225

**Published:** 2024-07-10

**Authors:** Jennifer I. Mejaes, Jacqueline Saenz, Chris O’Brien, Carina M. Pizzano, Ping-Yue Pan, David J. Barker

**Affiliations:** ^1^Department of Psychology, Rutgers, The State University of New Jersey, Piscataway, NJ, United States; ^2^Department of Neuroscience and Cell Biology, Robert Wood Johnson Medical School, Rutgers University, Piscataway, NJ, United States; ^3^Brain Health Institute, Rutgers University, Piscataway, NJ, United States

**Keywords:** Parkinson’s, synaptojanin1, stimulants, mesolimbic, dopamine

## Abstract

The *synaptojanin-1* (*SYNJ1*) gene is known to be important for dopamine-related disorders. Recent evidence has demonstrated that Synj1 deficient mice (*Synj1*^+/−^) have impairments in dopaminergic synaptic vesicular recycling. However, less is known about how *Synj1* deficits affect the mesolimbic system, reward processing, and motivated behavior. To examine the role of the *Synj1* gene in motivated behavior, we subjected male and female *Synj1*^+/−^ and *Synj1*^+/+^ mice to a battery of behavioral tests evaluating hedonic responses, effortful responding, and responses to psychomotor stimulants. We observed that *Synj1*^+/−^ mice exhibit few differences in reward processing and motivated behavior, with normal hedonic responses and motivated responding for sucrose. However, male but not female *Synj1*^+/−^ demonstrated an attenuated conditioned place preference for cocaine that could not be attributed to deficits in spatial memory. To further understand the dopamine signaling underlying the attenuated response to cocaine in these mutant mice, we recorded nucleus accumbens dopamine in response to cocaine and observed that *Synj1*^+/−^ male and female mice took longer to reach peak dopamine release following experimenter-administered cocaine. However, female mice also showed slower decay in accumbens dopamine that appear to be linked to differences in cocaine-induced DAT responses. These findings demonstrate that *SYNJ1* deficiencies result in abnormal mesolimbic DA signaling which has not previously been demonstrated. Our work also highlights the need to develop targeted therapeutics capable of restoring deficits in DAT function, which may be effective for reversing the pathologies associated with *Synj1* mutations.

## 1 Introduction

Though not originally conceptualized as a genetic disorder, several genetic loci (PARK genes) have been identified to associate with familial Parkinson’s disease (PD) (Klein et al., 2009; Chung et al., 2011; Li et al., 2021). Many of these genes share common cellular and molecular signaling pathways leading to the dysregulation of dopamine signaling and dopamine neuron vulnerability (Dong-Chen et al., 2023). With broad effects on dopaminergic neurons, symptoms associated with most variations of PD include decreases in motivation and goal-directed behaviors (Pagonabarraga et al., 2015). Notably, the presence of nonmotor symptoms such as apathy, depression, or other forms of motivational disturbances have been documented to precede the first motor symptoms for as many as 36% of pre-treatment PD patients (Pont-Sunyer et al., 2015). Thus, a better understanding of the motivational features associated with PD may have important predictive value for both the presence and progression of the disease.

One of the PARK genes is *SYNJ1*, also known as PARK20, which encodes an important inositol phosphatase, synaptojanin1. *SYNJ1* is essential for a variety of cellular functions related to neurotransmission including synaptic vesicle recycling (Cremona et al., 1999; Harris et al., 2000; Mani et al., 2007; Pan et al., 2020). Our preliminary study demonstrated that, while homozygous deletion of *Synj1* leads to non-survival in mice, a heterozygous deletion of *Synj1* (*Synj1*^+/−^) in mice is associated with impaired motor coordination in aged mice, as well as a reduction in the number of striatal dopaminergic terminals (Pan et al., 2020). Additionally, these mice exhibit an increased accumulation of cortical and striatal α-synuclein, which is a hallmark of PD (Pan et al., 2020). While *SYNJ1* is most notably known as a PD gene (Krebs et al., 2013; Quadri et al., 2013; Olgiati et al., 2014; Kirola et al., 2016; Taghavi et al., 2018; Xie et al., 2019; Lesage et al., 2021), it has also been linked with several other mental illnesses associated with dopaminergic dysfunction, including autism (Wang et al., 2015), bipolar disorder (Saito et al., 2001; Kato et al., 2007), and schizophrenia (Arion et al., 2007; Rodríguez-López et al., 2018). Moreover, the dopamine system malfunction associated with PD and parkinsonism has also been associated with affective and motivational deficits such as apathy and a blunted reward sensitivity (Muhammed et al., 2016; Pagonabarraga and Kulisevsky, 2017). Taken together, these findings suggest a broader role for *SYNJ1* that includes potential deficits in motivated or goal-directed behaviors. Indeed, we recently discovered that cocaine exposure leads to a Synaptojanin1-sensitive dopamine transporter (DAT) internalization process, where *Synj1* influences DAT surface expression (Saenz et al., 2022). Nonetheless, a full characterization of the effects *Synj1* has on motivated behavior has yet to be conducted.

The goal of the current study was therefore to conduct a broad characterization of motivation in *Synj1*^+/−^ mice. This characterization included a comprehensive battery of tests ranging from locomotor and exploratory responses to tests of effort-related motivation and drug reward. We observed that *Synj1*^+/−^ mice did not show any deficits in exploratory behaviors, behavioral avoidance or effort-related choice. In contrast, we discovered that *Synj1* deficient male mice showed an increased motivation to obtain a sucrose reward and specific deficits in cocaine reward, which appear to be linked with differential DA kinetics in response to cocaine. These experiments highlight key mechanistic differences in the mesostriatal dopamine dynamics that occur as a result of *Synj1* mutations and may provide crucial insights that aid in the development or management of dopamine replacement therapies.

## 2 Methods

### 2.1 Subjects

For all behavioral tests, subjects consisted of a sex and age-matched cohort of *Synj1*^+/+^ and *Synj1*^+/−^ littermate mice [total *n* = 77: *Synj1*^+/+^ male (*n* = 24), *Synj1*^+/+^ female (*n* = 11), *Synj1*^+/−^ male (*n* = 29), *Synj1*^+/−^ female (*n* = 13)]. Mice were 6–8 months of age at the start of the experiments. Mice were first run as a balanced cohort of 10 females and 12 males, split evenly by genotype; a second cohort of male mice was subsequently added as a technical replicate used to confirm several outcomes that were unique to males. All animals were group housed in cages consisting of *Synj1*^+/+^ and *Synj1*^+/−^ mice, cages were randomly selected from the colony for behavioral testing. Mice were held in a temperature-controlled vivarium with a 12 h: 12 h light: dark cycle with dawn at 7 AM. All mice had *ad libitum* access to food and water throughout testing, unless otherwise noted below.

### 2.2 Behavioral assays

The behavioral assays were conducted in the same order for all mice, as follows: elevated plus maze & open field assay (day 1), sucrose preference test (SPT) (days 2–5), operant conditioning paradigm (days 6–20), progressive ratio-schedule task (days 21), a cocaine conditioned place (CPP) preference paradigm that included a drug primed reinstatement test (days 22–33). An additional separate cohort that was not subjected to any of the above assays was run through an object location memory task (1–8 days), that served as a control test for the cocaine conditioned place preference paradigm. For all testing, the apparatus was cleaned between groups with 70% ethanol. All behaviors were conducted during the light phase of the light–dark cycle, unless otherwise indicated.

#### 2.2.1 Elevated plus maze

**Figure 1:**
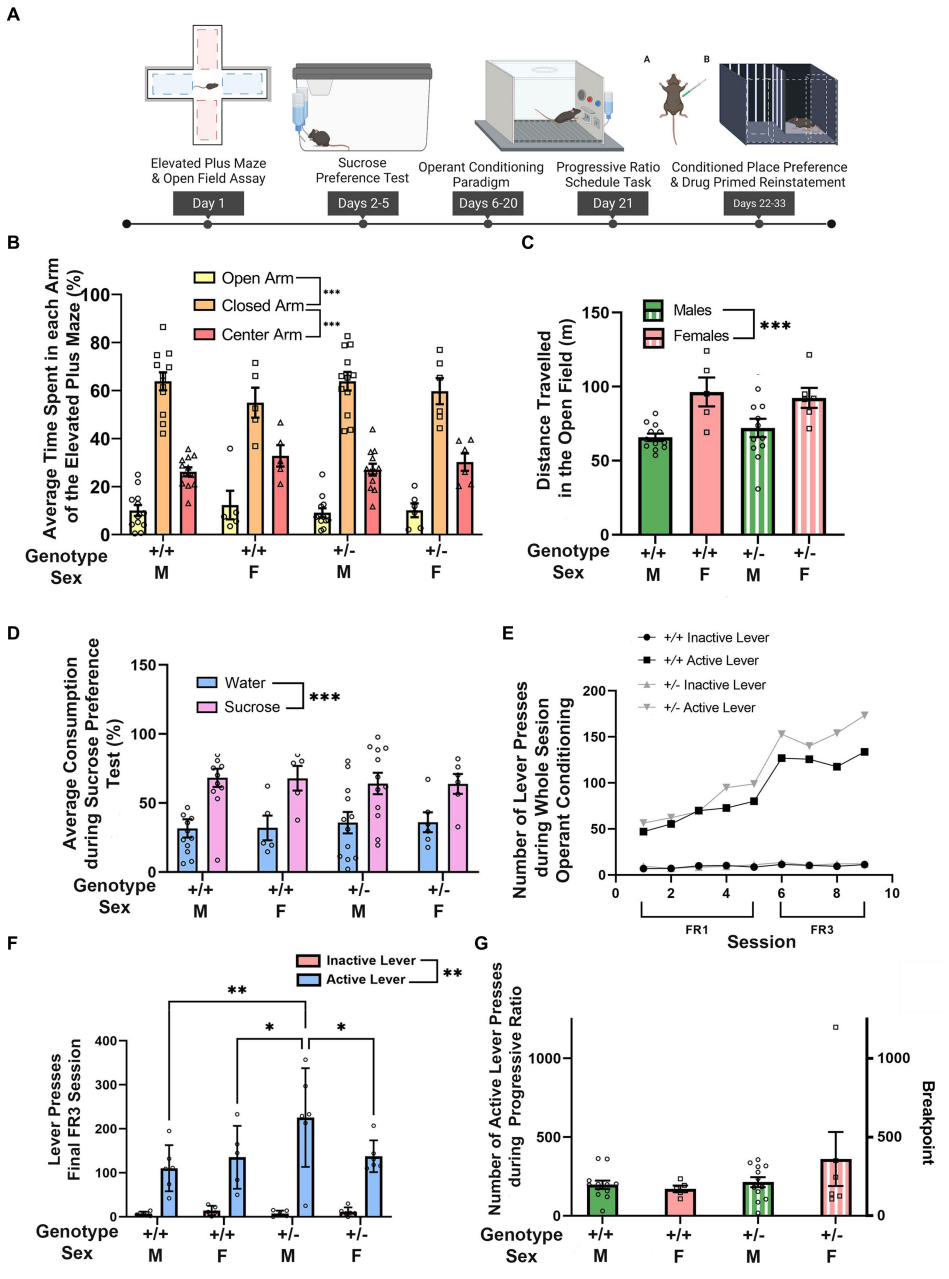
*Synj1* deficient mice show normal exploration, avoidance, and reward learning behaviors. **(A)** *Synj1*^+/+^ males (*n* = 12) and females (*n* = 5) as well as *Synj1*^+/−^ males (*n* = 12) and females (*n* = 6) were compared across a battery of tests for exploration, behavioral avoidance, hedonic responses to reward, and effort-related motivation. **(B)** All mice spent more time in the closed arm of the elevated plus maze with no differences between *Synj1*^+/−^ mice and *Synj1*^+/+^, nor across sexes. **(C)** In the open field, we observed greater locomotion in female mice as compared to males, but we did not observe any difference between *Synj1*^+/−^ and *Synj1*^+/+^ mice. **(D)** Both *Synj1*^+/−^ and *Synj1*^+/+^ mice preferred sucrose over water, showing a normal hedonic response to sucrose. **(E–G)** We did not observe any differences in reward learning between *Synj1*^+/−^ and *Synj1*^+/+^, **(F)** except during the last day of the FR3 schedule task where *Synj1*^+/−^ male mice, pressed the active lever significantly more compared to *Synj1*^+/+^ males, *Synj1*^+/+^ females as well as *Synj1*^+/−^ females. Moreover, all mice responded more on the active lever when compared to the inactive lever **(E)** and exhibited similar breakpoints in a progressive ratio task **(G)**. Values plotted are means ± SEM. ^***^p < 0.001.

*Synj1*^+/+^ males (*n* = 12) and females (*n* = 5) as well as *Synj1*^+/−^ males (*n* = 12) and females (*n* = 6) were run through the elevated plus maze (EPM). This maze consists of four arms that are joined in two perpendicular pairs, forming a plus-sign. Each arm was 30 cm long and 5 cm wide. Two of the arms are open and the other two are enclosed by 15 cm high walls (Figure 1A). Prior to the start of the assay, the mice were habituated in their home cages for 30 min. Following the habituation, each mouse was placed at the junction of the four arms of the EPM and allowed to explore the maze for 5 min. The mouse’s location in the maze, including entries into each arm, time spent in each arm and locomotor activity were recorded via video tracking software (ANYmaze).

#### 2.2.2 Open field

*Synj1*^+/+^ males (*n* = 12) and females (*n* = 5) as well as *Synj1*^+/−^ males (*n* = 12) and females (*n* = 6) were run through the open field (OF). The OF consisted of a 40 cm tall by 100 cm^2^ box. Mice were habituated in their home cages, prior to the start of the assay for 30 min. Following the habituation, each mouse was placed at the center of the field and their locomotor activity was recorded (ANYmaze) for 30 min.

#### 2.2.3 Sucrose preference test

*Synj1*^+/+^ males (*n* = 12) and females (*n* = 5) as well as *Synj1*^+/−^ males (*n* = 12) and females (*n* = 6) mice were singly housed in a home cage and acclimated to two 15 mL sipper bottles of water (Drinko Bottles; Amuza) overnight (Figure 1A). One bottle was then replaced with a 1% sucrose solution and the consumption of both water and sucrose were measured for a period of 2 days. Both bottles were weighed twice a day (morning and late afternoon) and refilled as necessary. Additionally, the position of bottles was switched each morning during weighing to combat side bias. The total sucrose or water consumed was calculated by using the amount (mL) of sucrose or water consumed over the total amount (mL) of consumption for both sucrose and water to normalize for differences in consumption.

#### 2.2.4 Operant responding for sucrose and progressive ratio-schedule task

*Synj1*^+/+^ males (*n* = 12) and females (*n* = 5) as well as *Synj1*^+/−^ males (*n* = 12) and females (*n* = 6) were trained in an operant conditioning task for a sucrose reward and subsequently tested them on a progressive ratio task. Before training mice in the operant boxes, all mice underwent food deprivation for approximately 1 week until they reached ~85% of their free-feeding weight.

The mice were trained and tested in standard mouse operant chambers (ENV-307A, Med Associates, Fairfax, VT) (Figure 1A). The operant chambers were controlled by an interface connected to a computer running the Med-PC IV software. The operant chambers contained two retractable levers and one retractable sipper containing 8% sucrose.

Mice were first habituated to the sipper before being trained with an autoshaping program where the extension of the right lever was associated with the extension of the sipper containing 8% sucrose. Mice were then transitioned to an FR-1 schedule until they reach an acquisition criterion of >30 active lever presses a day for at least 5 consecutive days. The mice were then trained on an FR-3 schedule for 4 days. Following FR-3 training, mice were run on a progressive ratio test for 1 day. The progressive ratio-schedule incremented linearly by 3 upon the completion of each successful criterion (i.e., 1, 4, 7 … *n* − 1 + 3) until the mice ceased to respond.

#### 2.2.5 Cocaine conditioned place preference paradigm

*Synj1*^+/+^ males (*n* = 12) and females (*n* = 5) as well as *Synj1*^+/−^ males (*n* = 12) and females (*n* = 6), were run through the cocaine conditioned place preference paradigm; one male mouse failed to complete the post-test, but was included for other analyses.

The CPP apparatus (Stoelting Co.) was made up of a three-compartment chamber consisting of (1) a large zone with striped walls, (2) a large zone with black walls and a grated metal floor, and (3) a small connecting chamber between the striped and black zones. A camera was secured ~1 meter above each box and was connected to a computer running the ANY-Maze software.

**Figure 2:**
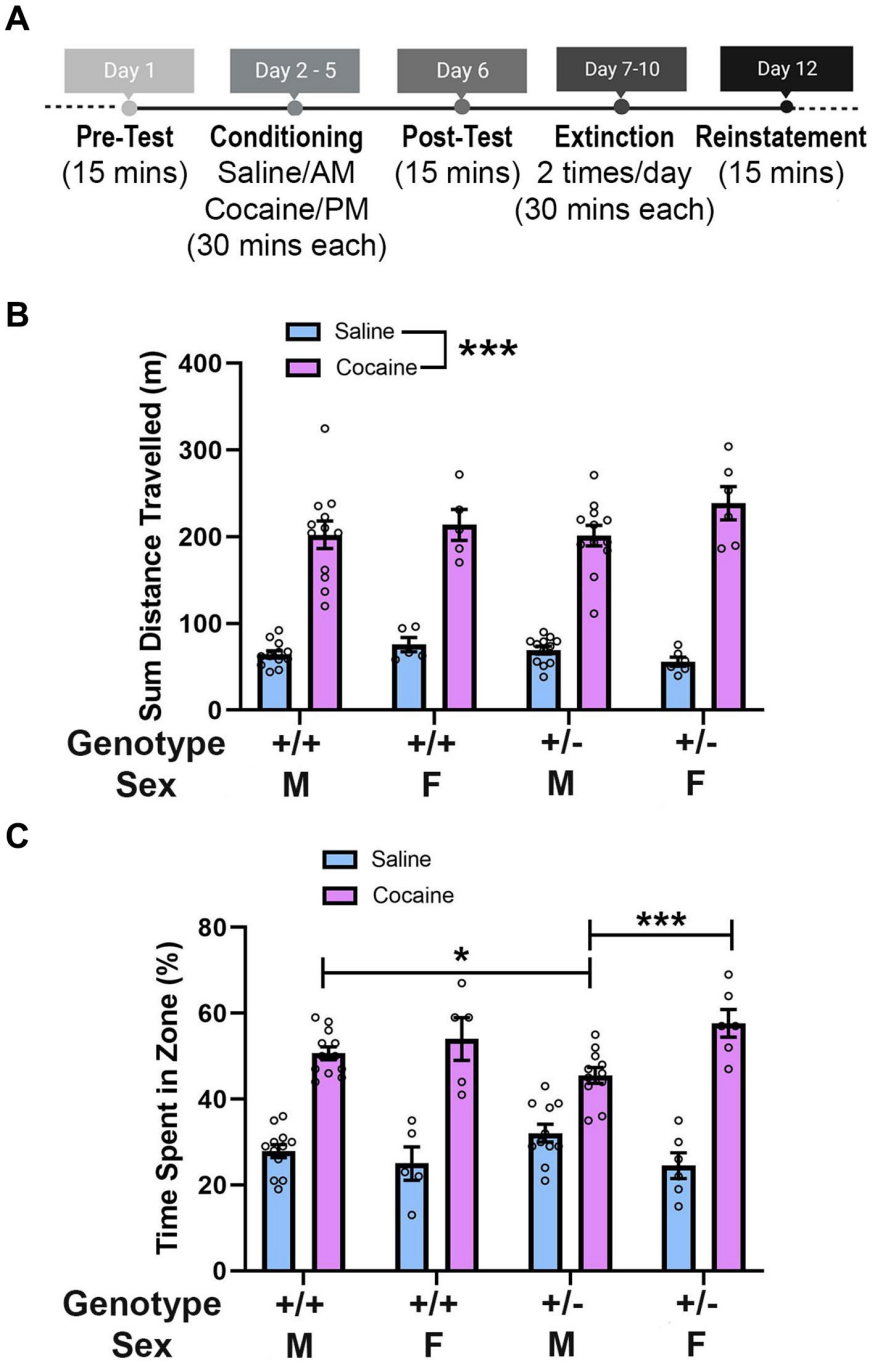
*Synj1* deficient male mice show weaker responses to the rewarding effects of cocaine. **(A)** *Synj1*^+/+^ males (*n* = 12) and females (*n* = 5) as well as *Synj1*^+/−^ males (*n* = 12) and females (*n* = 6), were run through the cocaine conditioned place preference paradigm conditioning days and *Synj1*^+/+^ males (*n* = 12) and females (*n* = 5) as well as *Synj1*^+/−^ males (*n* = 11) and females (*n* = 6) were run through the cocaine conditioned place preference paradigm post-test, to examine how *Synj1*^+/−^ and *Synj1*^+/+^ mice differ in their response to cocaine. **(B)** Throughout the conditioning days, all mice traveled more following injections of cocaine, as compared to saline, yet no differences were observed between *Synj1*^+/−^ and *Synj1*^+/+^ mice, nor across sexes. **(C)** During the post-test, all mice showed a preference for the cocaine paired chamber as compared to the saline paired chamber. However, male *Synj1*^+/−^ mice exhibited a weaker preference for cocaine, compared to both *Synj1*^+/+^ males as well as *Synj1*^+/−^ females. Values plotted are means ± SEM. ^*^p < 0.05 and ^***^p < 0.001.

Prior to the start of the assay, the mice were habituated to the CPP boxes for 45 min. The day after habituation, 1 pretest session was conducted. Subsequently, 4 conditioning sessions took place over 4 consecutive days. Conditioning sessions employed a biased design, such that cocaine was paired with the side least preferred during the pre-test. On each pairing day, mice were injected in the morning with 0.3 mL of 0.9% saline (i.e., physiological saline; i.p.) and isolated in the saline-paired chamber for 30 min. In the afternoon, mice were injected with 15 mg/kg of cocaine dissolved in saline (i.p.) and isolated in the cocaine-paired chamber for 30 min. Following the conditioning sessions, mice were given one 15 min post-test (Figure 2A).

#### 2.2.6 Object location memory task

**Figure 3:**
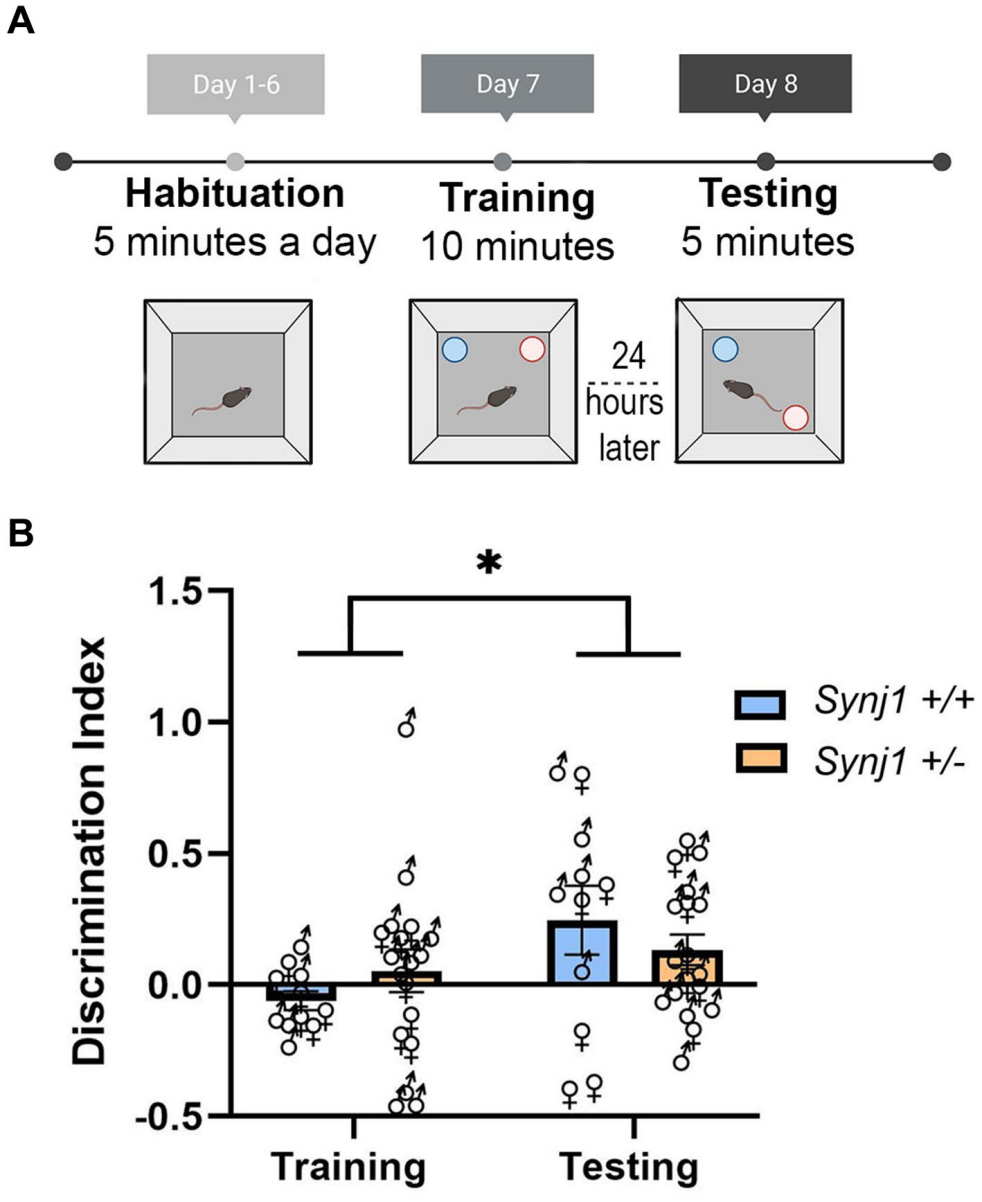
*Synj1*^+/−^ mice do not have any deficits in their spatial memory. **(A)** A naïve cohort of age-matched *Synj1*^+/+^ males (*n* = 5) and females (*n* = 6) as well as *Synj1*^+/−^ males (*n* = 11) and females (*n* = 7), were run through the object location memory task (OLM). **(B)** When examining the discrimination index, it was found that all mice, regardless of genotype, had a significantly higher discrimination index during the testing day as compared to the training day, where they spent more time interacting with the displaced object. This indicates that *Synj1*^+/−^ mice do not have diminished spatial memory, compared to *Synj1*^+/+^ mice [*F* (1,25) = 4.57, *p* < 0.05]. Values plotted are means ± SEM. ^*^p < 0.05.

A naïve cohort of age-matched *Synj1*^+/+^ males (*n* = 5) and females (*n* = 6) as well as *Synj1*^+/−^ males (*n* = 11) and females (*n* = 7), were run through the object location memory task (OLM). This task was used to assess spatial memory to ensure that differences observed cocaine-conditioned place preference paradigm could not be explained by deficits in spatial memory (Vogel-Ciernia and Wood, 2014). The OLM apparatus (Stoelting Co.) consisted of a 40 cm tall by 100 cm^2^ box (Figure 3A). The two objects used to test the long-term memory potential of the *Synj1*^+/−^ mice as well as their littermate controls (*Synj1*^+/+^) were a can and bottle of similar size but differing visual features, shapes, and textures. All mice were habituated to the empty boxes for 5 min a day for 6 days in a row. On day 7, a training session was conducted where the mice were placed into the same boxes, but with the objects added into the boxes close to one wall (Figure 3A). Mice were allowed to explore the box and objects for 10 min. Twenty-four hours later, mice were then placed back into the box with the objects, but with the can moved to a new location for a 5 min test. For all sessions, the behavioral tracking software ANY-maze was used to record the animal’s locomotive behaviors for the duration of the session.

### 2.3 Nucleus accumbens fiber-photometry dLight recordings

#### 2.3.1 Surgeries and injections

*Synj1*^+/+^ males (*n* = 7) and females (*n* = 6) as well as *Synj1*^+/−^ males (*n* = 6) and females (*n* = 7) were initially anesthetized with 1–5% isoflurane and then maintained using 1–3% isoflurane thereafter. Perioperative Carprofen (5 mg/kg) and Baytril (5 mg/kg) were administered subcutaneously along with a local injection of bupivacaine (2.5 mg/kg).

**Figure 4:**
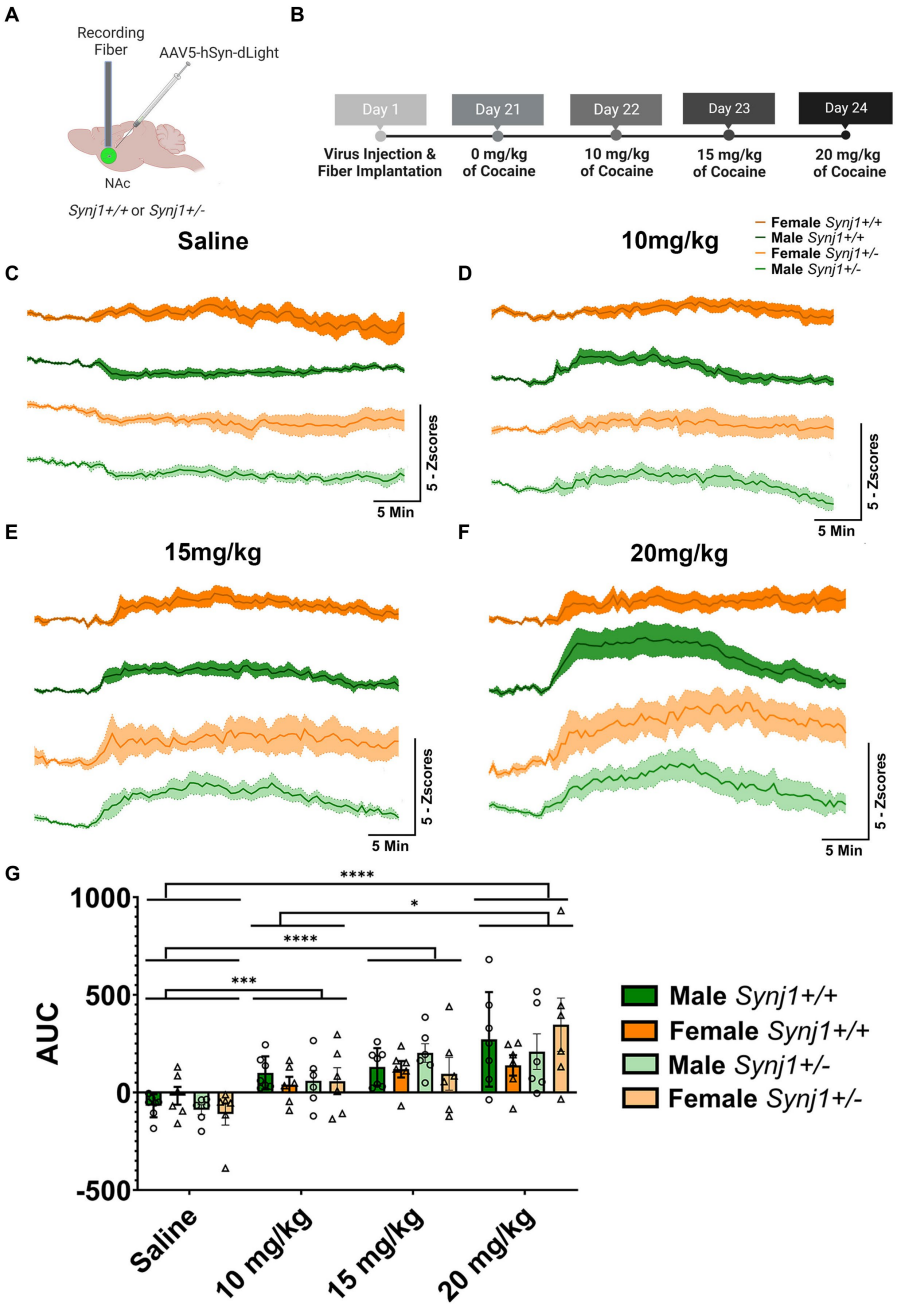
*Synj1* deficits do not alter the total amplitude of dLight signals. **(A)** A naïve cohort of *Synj1*^+/+^ males (*n* = 7) and females (*n* = 6) as well as *Synj1*^+/−^ males (*n* = 6) and females (*n* = 7) were injected with the dLight dopamine sensor in the NAc, **(B)** which was used to evaluate dopamine signaling in this brain region across varying doses of cocaine, using a fiber-photometry set-up. When all mice were injected with **(C)** saline, a modest decrease in dLight signaling was observed. Additionally, a dose-dependent increase in dLight signal was observed following **(D)** 10, **(E)** 15, or **(F)** 20 mg/kg of cocaine. **(G)** The amplitude of the dLight signal did not differ between *Synj1*^+/−^ and *Synj1*^+/+^ mice. However, compared to saline the total amplitude of dLight signals were greater at all cocaine doses, with the highest dose of cocaine (20 mg/kg) being significantly enhanced when compared to the lowest dose. Values plotted area means ± SEM. ^*^p < 0.05, ^***^p < 0.001, and ^****^p < 0.0001.

After being fixed in a stereotaxic frame, a midline incision was made along the scalp and the skull leveled. The viral vector AAV5-hSyn-dLight1.2-WPRE (1.6 × 10^13^ GC/mL, Addgene 1110686) was injected (300 nL at 50 nL/min) targeting the NAc (AP: +1.25; ML: + 0.75; DV: −4.55) using a Nanofil syringe (World Precision Instruments) and micro-infusion controller (Smart Touch, World Precision Instruments). The needle was left in place for 10 min. A fiber optic (Doric Lenses) was then implanted 100 μm above the injection site and secured to the skull with three jewelers screws and dental acrylic containing 5% carbon (Figure 4A). Following surgery, the mice were given postoperative care for a minimum of 3 days, including daily injections of Carprofen (5 mg/kg) and Baytril (5 mg/kg).

#### 2.3.2 Fiber-photometry recordings

For all recordings, dLight was excited at two wavelengths (490 nm for the dLight signal and 405 nm as an isosbestic control) by amplitude modulated signals from two light-emitting diodes (Doric) connected to an optical minicube (Doric) that was coupled into the implanted optic fiber via a fiber optic patch cord. Signals emitted from dLight and the isosbestic control channel then returned through the same optic fiber and were acquired using a photodetector integrated into the minicube. Signals were then digitized at 1 kHz, and then recorded by a real-time signal processor (RZ2; Tucker Davis Technologies) running the Synapse software suite. Analysis of the resulting signal was then performed using pMAT (Bruno et al., 2021) or custom-written MATLAB scripts.

**Figure 5:**
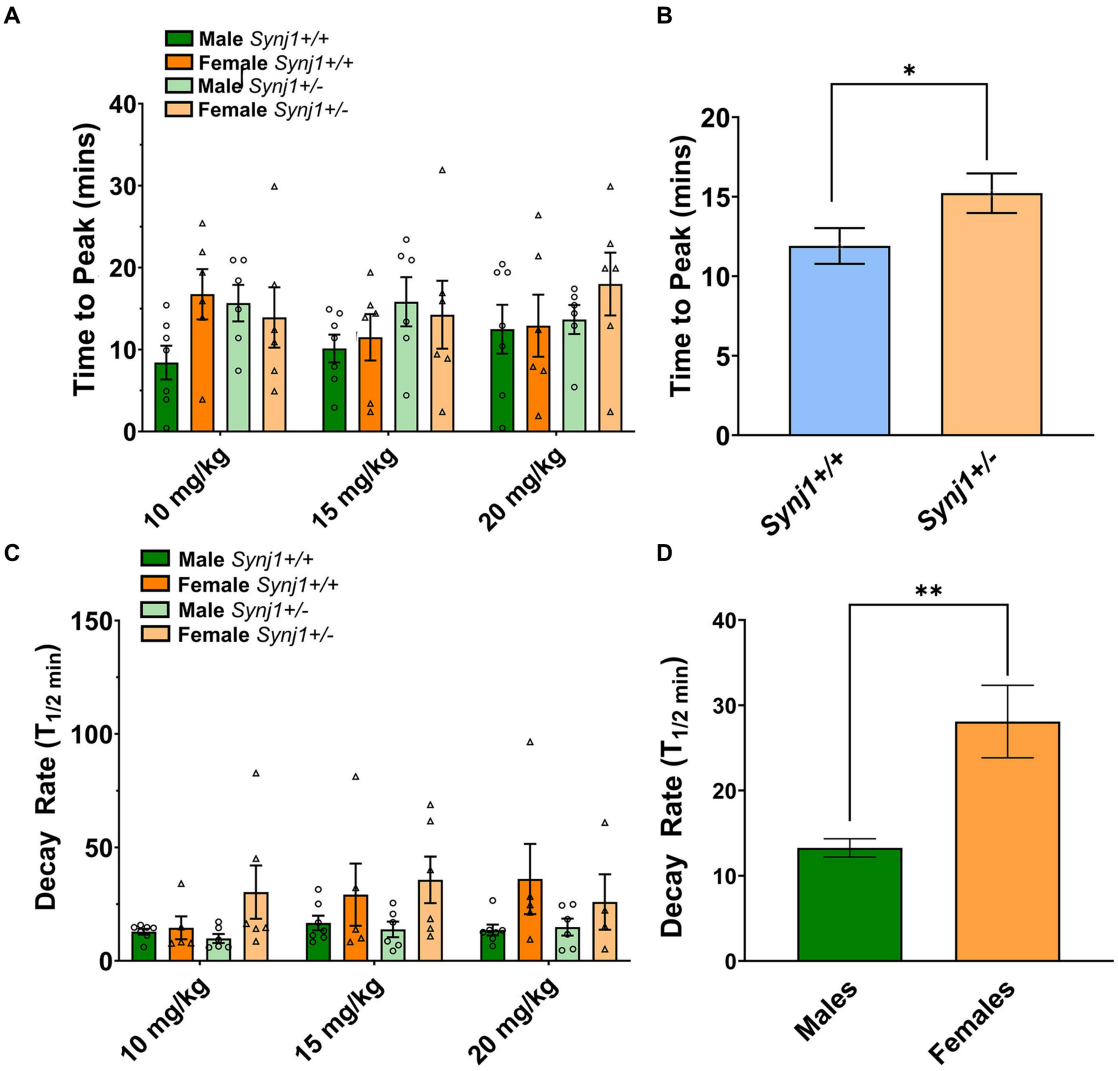
Repeated *in-vivo* cocaine exposure increases DAT expression in male *Synj1*^+/+^, but not in male *Synj1*^+/−^ mice. **(A)** To conduct western blot analyses and quantify DAT levels, the midbrain, and striatum were extracted from a cohort of littermate *Synj1*^+/+^ and *Synj1*^+/−^ mice. Half the mice received an injection of saline [*Synj1*^+/+^ males (*n* = 8), *Synj1*^+/−^ males (*n* = 8), *Synj1*^+/+^ females (*n* = 4), female *Synj1*^+/−^(*n* = 4)], and the other half received an injection of cocaine [*Synj1*^+/+^ males (*n* = 7), *Synj1*^+/−^ males (*n* = 9), *Synj1*^+/+^ females (*n* = 5), *Synj1*^+/−^ females (*n* = 5)]. **(B,C)** In the midbrain DAT expression was significantly enhanced in the cocaine-treated male *Synj1*^+/+^ mice compared to the female *Synj1*^+/+^. **(D,E)** Moreover, in the striatum, a similar trend was observed where DAT expression was significantly elevated in the cocaine-treated male *Synj1*^+/+^ mice compared to the female *Synj1*^+/+^ and saline-treated male *Synj1*^+/−^ mice compared to their female *Synj1*^+/−^ counterparts. Values plotted are means ± SEM. ^*^p < 0.05 and ^***^p < 0.001.

The fiber-photometry recordings were conducted in an open field (described above). dLight responses were taken across an ascending series of cocaine doses [0 (saline), 10, 15, and 20 mg/kg] with one injection per day over a period of 4 days (Figures 4,5).

#### 2.3.3 Histology

Mice were anesthetized with isoflurane and transcardially perfused with a 0.1 M phosphate buffer, followed by 4% paraformaldehyde. After the brains were extracted, they were post-fixed in paraformaldehyde overnight. After post-fixation, the brains were transferred to an 18% sucrose solution overnight until they sank. The brains were then frozen in powdered dry ice. After being frozen all brains were then stored at −80°C for cryo-sectioning. The coronal brain sections were sliced at 40 μm thickness on the cryostat and mounted onto charged slides (Superfrost Plus). Slides were then imaged at 20× magnification using a Keyence BZX800 microscope to validate fiber optic placements.

### 2.4 Western blot analysis

**Figure 6:**
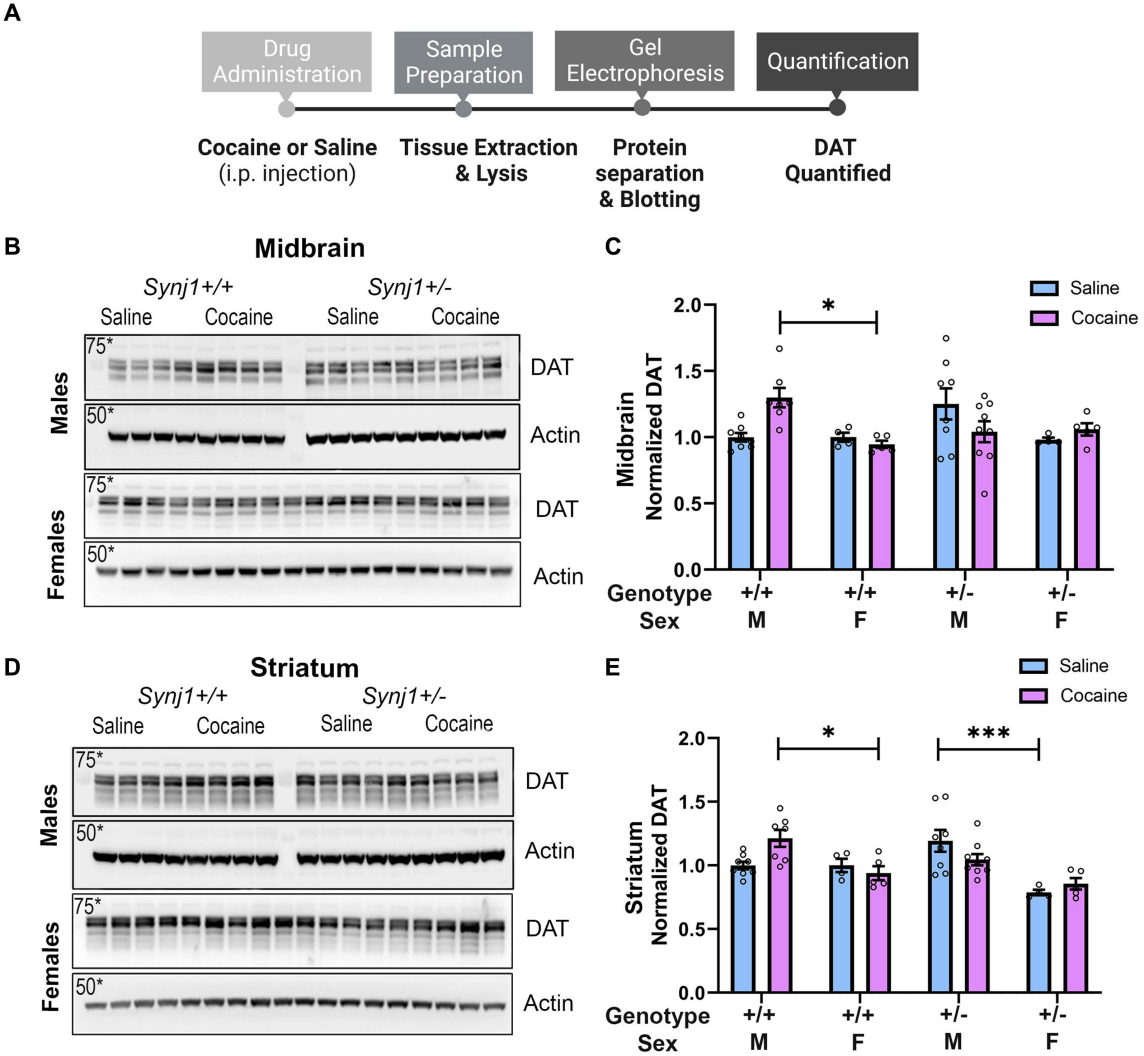
*Synj1* deficits alter striatal DA kinetics in males and females when treated with cocaine. We modeled the latency for dopamine signals to peak (*T*_peak_), as well as the half-decay time (*T*_1/2_). **(A,B)** Our modeling of dopamine kinetics indicated that the *T*_peak_ was significantly longer in all *Synj1*^+/−^ mice as compared to *Synj1*^+/+^ controls. **(C,D)** Moreover, in terms of the *T*_1/2_, no differences between genotypes were observed, however, all females appeared to have a longer *T*_1/2_ compared to all males. Values plotted are means ± SEM. ^*^p < 0.05 and ^**^p < 0.01.

An additional cohort of animals at the age of ~10 months that was not run through any behavioral testing were i.p. injected with either saline [*Synj1*^+/+^ males (*n* = 8), *Synj1*^+/−^ males (*n* = 8), *Synj1*^+/+^ females (*n* = 4), female *Synj1*^+/−^ (*n* = 4) or cocaine] [*Synj1*^+/+^ males (*n* = 7), *Synj1*^+/−^ males (*n* = 9), *Synj1*^+/+^ females (*n* = 5), *Synj1*^+/−^ females (*n* = 5)] on the same schedule as the CPP test (4 daily injections) to analyze the effects of these treatments on DAT levels in the midbrain and striatum (Figure 6A).

#### 2.4.1 Sample preparation

All animals were sacrificed at 30 min after cocaine/saline treatment and several regions of the brain were extracted including the midbrain and striatum. The extracted tissue was immediately frozen on dry ice and kept at −80°C until ready to lyse. When the tissue was ready to lyse, it was defrosted on ice and an ice-cold lysis buffer was added. The lysis buffer contained 50 mM Tris-HCl, 150 mM NaCl, 1% Triton X100, supplemented with a cocktail of protease inhibitor (Roche REF # 11836170001) and phosphatase inhibitor (Roche REF 04906837001), and was added while a motorized pestle was used to grind up the tissue. Tissue lysates were placed on a rotor for 30 min at 4°C to allow the lysing to continue. After 30 min of incubation on the rotor, the tissue was centrifuged at 4°C for 30 min at 15,000 rpm. The supernatant was then collected and kept at −80°C. and quantified using the Pierce BCA Protein Assay kit (Thermo Fisher 23227) to determine the protein concentration. After determining the protein concentration of each sample, the samples were then diluted into NuPAGE LDS sample buffer (Thermo Fisher NP007) containing 50 mM final concentration of a reducing agent (Dithiothreitol or DTT) and water.

#### 2.4.2 Gel electrophoresis and blotting

Prior to loading, samples were denatured at 70°C for 10 min. Twenty micrograms of each protein sample was loaded onto polyacrylamide Invitrogen NuPAGE 4–12% Bis-Tris gels. After separating the proteins, they were transferred to a membrane using a wet transfer method. Following this transfer, the membrane was first blocked with 5% BSA for 1 h, followed by incubation with the primary antibody overnight, with anti-DAT (1:1000, Millipore AB2231 antibody). The same membrane was blotted for β-actin as a loading control (1:1000, cell signaling 3700S). Protein was detected using the labeled antibody horseradish peroxidase (HRP).

For validation of the antibody specificity, the results for the striatum were replicated using another KO validated antibody (Thermo Fisher, MA5-24796; data not shown) (Russo et al., 2023).

#### 2.4.3 Quantification

Protein bands were analyzed using ImageJ. The most prominent DAT band was detected at ~80 kDa, and it was normalized to the corresponding β-actin band for each sample, which is further normalized to the mean of the *Synj1*^+/+^ saline treated group. Altogether, 2–3 technical replicates were performed for each sample to obtain the average. The same analyses were performed for the midbrain and striatum.

### 2.5 Statistical analyses

One-way, two-way and three-way ANOVAs or generalized linear models were performed for each behavioral assay in SPSS^®^ or GraphPad Prism with genotype (*Synj1*^+/−^ and *Synj1*^+/+^) and sex as between-subjects factors. Homogeneity of variance and normality were confirmed for all data analyzed using ANOVAs. As needed, the Greenhouse–Geisser correction was applied to correct violations in the assumption of sphericity, and Sidak post-hoc corrections were used to control the familywise error rate. Due to violations in the assumption of normality, a generalized linear model was used for data from the cocaine-conditioned place preference experiment. Upon noting this singular violation, the results for all ANOVAs were examined and confirmed using generalized linear models but are presented as ANOVAs for simplicity.

## 3 Results

### 3.1 *Synj1* deficiency does not affect exploratory behaviors or behavioral avoidance

We first wanted to assess whether *Synj1*^+/−^ mice differed from *Synj1*^+/+^ controls by examining their exploratory behaviors as well as natural avoidance responses in the elevated plus maze and open field (Figure 1A). In the elevated plus maze, we observed that all mice spent more time in the closed arms than in the open arm [*t*(34) = 13.36, *p* < 0.001] or center zone [*t*(34) = 8.45, *p* < 0.001; Arm: *F* (2,62) = 124.01, *p* < 0.001; Figure 1B]. However, there were no differences between males and females, nor between *Synj1*^+/−^ and *Synj1*^+/+^ mice [sex (*F* (1,31) = 0.72, *p* = 0.40), genotype (*F* (1,31) = 0.21, *p* = 0.65), sex*genotype (*F* (1,31) = 0.01, *p* = 0.94), sex*arm (*F* (2,62) = 1.66, *p* = 0.20), genotype*arm (*F* (2,62) = 0.21, *p* = 0.72), sex*genotype*arm (*F* (2,62) = 0.22, *p* = 0.71)].

Similarly, when examining exploratory behaviors in the open field, we did not observe any difference in locomotion between *Synj1*^+/−^ and *Synj1*^+/+^ mice. However, we did observe that female mice traveled a greater distance than males, irrespective of genotype [*t*(33) = 4.05, *p* < 0.001; Sex: *F* (1,31) = 16.44, *p* < 0.001; Figure 1C]. No additional effects of sex or genotype were observed [genotype (*F* (1,31) = 0.03, *p* = 0.86), sex*genotype (*F* (1,31) = 0.68, *p* = 0.42)].

### 3.2 *Synj1* deficient mice exhibit normal hedonic responses

We next sought to determine whether *Synj1*^+/−^ mice differed from *Synj1*^+/+^ controls in their hedonic responses to reward in a sucrose preference task (Figure 1A). In the sucrose preference test we observed that, on average, male mice drank more than female mice, consistent with their differences in body weight, but did not observe any obvious differences in liquid consumption across genotypes. We therefore compared the relative percentage of sucrose and water for each subject to control for either individual or sex differences in overall liquid consumption. When comparing *Synj1*^+/−^ and *Synj1*^+/+^ mice, we observed that all mice exhibited a strong preference for the sucrose bottle over water [*t*(34) = 3.78, *p* < 0.001; Bottle: *F* (1,31) = 14.27, *p* < 0.001; Figure 1D]. However, we did not observe any differences in the proportion of sucrose consumed between *Synj1*^+/−^ and *Synj1*^+/+^ mice, nor any differences across sexes [sex: (*F* (1,31) = 0.01, *p* = 1.00); genotype: (*F* (1,31) = 0.01, *p* = 1.00); sex*genotype: (*F* (1,31) = 0.01, *p* = 1.00); sex*bottle: (*F* (1,31) = 0.01, *p* = 0.97); genotype*bottle: (*F* (1,31) = 0.24, *p* = 0.63); sex*genotype*bottle: (*F* (1,31) = 0.01, *p* = 0.99)].

### 3.3 *Synj1* deficient mice exhibit normal reward learning, while *Synj1* deficient male mice demonstrated an increased effort-related motivation during the last day of the FR3 schedule task

Additionally, we examined reward learning and effort-related motivation by training mice to lever-press for access to a liquid sucrose reward using a fixed ratio paradigm before testing in a progressive ratio task (Figure 1A). During operant training, both *Synj1*^+/−^ and *Synj1*^+/+^ mice learned at a similar rate [Figure 1E; sessions 1–9: all *t*(*34*) *≥* 6.06, all *p* < 0.001; Lever*Session: *F* (8,272) = 23.25, *p* < 0.001]. Moreover, all mice responded more during the FR3 schedule (sessions 6–9) than the FR1 schedule (sessions 1–5), consistent with the increased response requirement from one to three responses [Figure 1E Schedule: *F* (1,31) = 37.11, *p* < 0.001; schedule*genotype (*F* (1,31) = 0.09, *p* = 0.77); schedule*sex (*F* (1,31) = 0.61, *p* = 0.44); sex*genotype (*F* (1,31) = 0.12, *p* = 0.73); schedule*genotype*sex (*F* (1,31) = 2.15, *p* = 0.15)]. During the last day of the FR3 schedule task all mice, regardless of sex or genotype pressed the active lever significantly more than the inactive lever [Figure 1F Lever: F (1,19) = 92.19, *p* < 0.0001; lever*sex*genotype interaction: F (3,19) = 3.174, *p* < 0.05]. However, we also observed that Synj1^+/−^ male mice, pressed the active lever significantly more than *Synj1*^+/+^ males [t(38) = 3.8, *p* < 0.05], *Synj1*^+/+^ females [t(38) = 2.84, *p* < 0.05] as well as Synj1^+/−^ females [*t*(38) = 2.9, *p* < 0.05].

Following operant training, we examined effortful responding in the progressive ratio task by determining the breakpoint for each mouse. The breakpoint represents the maximal number of responses resulting in the administration of sucrose before the cessation of responding. We did not observe any differences in the breakpoint between *Synj1*^+/−^ and *Synj1*^+/+^ mice, nor between males and females (Figure 1G) [sex (*F* (1,31) = 0.76, *p* = 0.39); genotype (*F* (1,31) = 2.22, *p* = 0.15); sex*genotype interaction (*F* (1,31) = 1.56, *p* = 0.22)].

### 3.4 *Synj1* deficient male mice show weaker responses to the rewarding effects of cocaine

We next sought to examine how *Synj1*^+/−^ and *Synj1*^+/+^ mice differed in their response to cocaine using a conditioned place preference paradigm (Figure 2A). We first determined whether there were differences in locomotion between *Synj1*^+/−^ and *Synj1*^+/+^ male or female mice following injections of saline or cocaine. We observed that all mice traveled more following injections of cocaine, as compared to saline [*F* (1,31) = 341.7, *p* < 0.0001; Figure 2B]. No differences were observed between *Synj1*^+/−^ and *Synj1*^+/+^ mice, nor across sexes [Sex: *F* (1,31) = 1.37, *p* = 0.25; Genotype: (*F* (1,31) = 0.05, *p* = 0.82); Treatment*Sex: (*F* (1,31) = 2.55, *p* = 0.12); Treatment*Genotype: (*F* (1,31) = 1.48, *p* = 0.23); Sex*Genotype: (*F* (1,31) = 0.001, *p* = 0.98); Treatment*Sex*Genotype: (*F* (1,31) = 2.50, *p* = 0.12)].

Following conditioning, mice showed a preference for the cocaine paired chamber as compared to the saline paired chamber [*X*^2^ (1) = 217.70, *p* < 0.001]. However, we also observed that male *Synj1*^+/−^ mice exhibited a weak preference for cocaine and found specific differences when compared to both *Synj1*^+/+^ males as well as *Synj1*^+/−^ females [Figure 2C; all *t*(34) > 2.00, all *p* < 0.05; Generalized Linear Model, sex*genotype*zone interaction: *X*^2^ (1) = 4.35, *p* < 0.05]. No additional effects were found [sex (*X*^2^ (1) = 0.06, p = 0.80), genotype (*X*^2^ (1) = 0.09, *p* = 0.76), sex*genotype (*X*^2^ (1) = 0.43, *p* = 0.51), genotype*zone (*X*^2^ (1) = 0.58, *p* = 0.45)].

### 3.5 *Synj1*^+/−^ male mice’s weaker cocaine place preference is not due to deficits in their spatial memory

Differences in place conditioning can result either from differences in reward processing or from differences in spatial learning for locations in the conditioning chamber. To exclude the possibility that *Synj1*^+/−^ male mice may have deficits in spatial memory, we examined the contextual memory of a naive cohort of *Synj1*^+/−^ and *Synj1*^+/+^ mice in an object location memory task (OLM) (Figure 3A) using a previously described protocol (Vogel-Ciernia and Wood, 2014). This task allows animals to briefly learn the spatial locations of two objects before one object is displaced 24 h later. The basic hypothesis is that mice with intact spatial memory will spend more time exploring the displaced object. The time spent exploring the displaced object is calculated by the discrimination index (DI): displaced object (s) − static object (s)/(displaced object (s) + static object (s)). For this index, positive values indicate more time spent investigating the displaced object, while negative values indicate more time exploring the static object during the testing day. When examining the discrimination index across the training and testing day, we did not observe any effect of genotype [*F* (1,27) < 0.0001, *p* = 0.99; Figure 3B], nor any genotype × day interaction [*F* (1,27) = 1.40, *p* = 0.25; Figure 3B]. However, we did observe that all mice regardless of genotype, had a higher discrimination index during the testing day vs. the training day [*F* (1,25) = 4.57, *p* < 0.05; Figure 3B]. This suggests that all mice interacted with the displaced object significantly more during testing than training, allowing us to infer that Synj1^+/−^ mice do not have any deficits with their spatial memory.

### 3.6 *Synj1*^+/−^ mice show delayed peak dopamine responses in the nucleus accumbens compared to littermate controls

We next examined transmission in the Nucleus Accumbens (NAc) of *Synj1*^+/−^ and *Synj1*^+/+^ male and female mice across escalating doses of cocaine using the DA-specific biosensor dLight 1.2 (Figures 4A,B). In both *Synj1*^+/+^ and *Synj1*^+/−^ mice, we observed modest decreases in dLight signals following an injection of saline. In contrast, we observed a dose-dependent increase in dLight signals following injections of 10, 15, and 20 mg/kg of cocaine (Figures 4C–F). When compared to saline, the total amplitude of dLight signals were greater at all cocaine doses [Figure 4G; Dose: *F* (1.75,36.7) = 20.93, *p* < 0.0001; Saline vs. 10, 15, or 20 mg/kg of cocaine, all *p* < 0.05]. However, no effect for sex nor genotype was observed [Figure 4G; Sex: *F* (1,21) = 0.03, *p* > 0.05; Genotype: *F* (1,21) = 0.2, *p* > 0.05]. Additionally, the dLight signals observed at the highest dose of cocaine (20 mg/kg) were significantly enhanced when compared to the lowest dose (20 mg/kg vs. 10 mg/kg, *p* < 0.01, Figure 4G).

In contrast, when examining the traces, notable differences in the kinetics between *Synj1*^+/−^ and *Synj1*^+/+^ mice, as well as male and female mice were observed (Figures 5A–D). We, therefore, modeled the latency for dopamine signals to peak (*T*_peak_), as well as the half-decay time (*T*_1/2_). Our modeling of dopamine kinetics indicated that the *T*_peak_ was significantly longer in all *Synj1*^+/−^ mice as compared to *Synj1*^+/+^ controls [Genotype: *F* (1,23) = 4.3, *p* < 0.05]. Moreover, in terms of the *T*_1/2_, while we did not observe any differences between genotypes [Genotype: *F* (1,23) = 0.08, *p* > 0.05], we did see an effect of sex [Sex: *F* (1,58) = 11.26, *p* < 0.01] with all females having a longer *T*_1/2_ compared to all males.

### 3.7 Male *Synj1*^+/−^ male mice show impaired adaptive changes in DAT expression

Abnormal cocaine-dLight responses in the *Synj1*^+/−^ mice suggested maladaptive DAT expression or function, which has been shown in our previous study as well as in another Synj1 mutant mouse (Cao et al., 2017; Saenz et al., 2022).

To examine the effects of cocaine (15 mg/kg/day) on DAT expression we i.p. injected a cohort of male and female *Synj1*^+/+^ and *Synj1*^+/−^ mice with saline or cocaine, for 4 consecutive days and then collected both the midbrain and striatum to test for cocaine-induced differences in DAT (Figure 6A). Our western blot analysis suggested that in the midbrain, an effect of sex, treatment, and genotype was seen [Figures 6B,C; Sex: *F* (1,42) = 6.9, *p* < 0.05; Treatment*Genotype*Sex: *F* (1,42) = 7.76, *p* < 0.01] where the DAT expression was significantly enhanced in the cocaine-treated male *Synj1*^+/+^ mice compared to the female *Synj1*^+/+^ (Figure 6C; *p* < 0.05). Moreover, in the striatum, a similar trend was observed (Figure 6D; Sex: *F* (1,42) = 24.24, *p* < 0.0001; Treatment*Genotype*Sex: *F* (1,42) = 7.74, *p* < 0.01), where DAT expression was significantly elevated in the cocaine-treated male *Synj1*^+/+^ mice compared to the female *Synj1*^+/+^ (Figure 6E; *p* < 0.05), and saline-treated male *Synj1*^+/−^ mice compared to their female *Synj1*^+/−^ counterparts (Figure 6E; *p* < 0.001). We interpret these findings to indicate that cocaine typically causes an increase in DAT expression for male mice, and that this effect does not occur in male *Synj1*^+/−^ mice, who already exhibit elevated levels of striatal DAT at baseline (saline condition). These effects may become even more pronounced following repeated cocaine use (Saenz et al., 2022).

## 4 Discussion

The synaptojanin1 (*Synj1*) gene has been shown to influence DA neurons and play an important role in vesicular recycling (Cremona et al., 1999; Harris et al., 2000; Mani et al., 2007; Chang-Ileto et al., 2011; Cao et al., 2017; Pan et al., 2020), but its effects on reward processing and motivation throughout the mesolimbic DA system have received far less attention. In the present study, we utilized *Synj1*^+/+^ and *Synj1*^+/−^ mice, intending to examine the effect that *Synj1* haploinsufficiency has on motivated behavior and reward processing. We observed that *Synj1*^+/−^ mice exhibit normal behavioral avoidance and locomotor behaviors as well as sucrose consumption. However, we observed differences in operant learning during the last day of the FR3 schedule task, with *Synj1*^+/−^ male mice exhibiting a greater sucrose seeking compared to their *Synj1*^+/+^ male and female counterparts. Moreover, male *Synj1*^+/−^ mice exhibited a weaker cocaine place preference compared to their male controls, and when compared to female *Synj1*^+/−^ mice. We empirically determined that the differences in cocaine preference were not related to deficits in their spatial memory, since no deficit in *Synj1*^+/−^ mice was observed. Instead, we discovered that the blunted response to cocaine reward exhibited by *Synj1* deficient male mice appears to be linked to differential DA kinetics following cocaine injections that are associated with different midbrain and striatal DAT expression. Specifically, we observed that both *Synj1*^+/−^ male and female mice took longer to reach peak DA responses following an injection of cocaine than their *Synj1*^+/+^ littermate controls. However, we also observed that DA responses took longer to decay in females than males, which may ultimately have protected normative cocaine conditioning in *Synj1*^+/−^ females. In contrast, we observed that male *Synj1*^+/−^ mice show elevated levels of DAT at baseline, and that these mice fail to show the cocaine-induced increases in DAT observed in *Synj1*^+/+^ males. When taken together, these data suggests that *Synj1* mutations produce specific differences in the mesostriatal DA signaling, which might be associated with DAT dysfunction. Understanding these mechanisms may help to better tailor treatments for Synj1-related disorders.

### 4.1 Reward processing in *Synj1*^+/−^ mice

Our prior work indicates that abnormal dopaminergic signaling in *Synj1* deficient mice may be due to aberrant synaptic vesicle recycling (Mani et al., 2007; Pan et al., 2020). Interestingly, it has been found that proteins involved in vesicular recycling may be involved in DAT trafficking as well (Gabriel et al., 2013), which supports our recent finding that *Synj1* directly influences the trafficking of DAT and cocaine-induced DAT internalization in midbrain axons (Saenz et al., 2022). One interesting but unexpected result of the current study was the observation that *Synj1* mutations do not lead to abnormal dopaminergic system function until pharmacologically challenged, suggesting that the DA system may function normally in younger mice except when pushed to physiological extremes. For example, during the cocaine conditioned place preference paradigm, *Synj1*^+/−^ male mice had a slightly weaker, yet significant, cocaine place preference compared to *Synj1*^+/+^ males and *Synj1*^+/−^ female mice—despite normal spatial memory. Additionally, when examining NAc DA transmission in *Synj1*^+/−^ and *Synj1*^+/+^ mice in response to escalating doses of cocaine, we observed a notable delay in the DA kinetics for all *Synj1*^+/−^ mice as compared to *Synj1*^+/+^ controls. Although *Synj1* mutations do not appear to initially lead to abnormal hedonic reward processing, the apparent deficits in dopaminergic processing that we observed in the NAc strongly implicates underlying dopaminergic system dysfunction that may become exacerbated in aged mice.

Elevated levels of baseline DAT are thought to catalyze dopaminergic neuronal degeneration, resulting in a delay in DA kinetics, and slower peak in DA levels in the NAc of *Synj1*^+/−^ mice. Indeed, there appears to be a direct association between synaptic dysfunction, neuronal degradation, and elevated levels of DAT in *SYNJ1* mice, where elevated DAT has been linked to a buildup of cytosolic DA and eventual synaptic oxidative damage and cell loss (Masoud et al., 2015; Cao et al., 2017). Thus, the effects of *Synj1* deficiency in males may be two-fold, with the elevated DAT leading to both an elongated response window for cocaine and the eventual deterioration of DA neurons due to abnormal DA signaling, recycling, and cytosolic buildup (Mavridis et al., 2011; Masoud et al., 2015; Mavridis, 2015; Saenz et al., 2022).

### 4.2 Considerations of *Synj1* deficiency and locomotor functions

A mutation in the *SYNJ1* gene has been attributed to parkinsonism and dopaminergic dysfunction (Krebs et al., 2013; Quadri et al., 2013; Olgiati et al., 2014; Kirola et al., 2016; Taghavi et al., 2018). Our prior work has shown that heterozygous deletion of *Synj1* (*Synj1*^+/−^ mice) is associated with impaired motor coordination as well as a reduction in the number of striatal dopaminergic terminals (Pan et al., 2020). However in our current study, all mice regardless of sex or genotype, exhibited normal locomotor, exploratory and avoidance behaviors compared to their controls. These normal locomotive behaviors were also demonstrated in all *Synj1*^+/−^ mice, even though they displayed higher baseline levels of DAT expression in their midbrain and striatum compared to *Synj1*^+/+^ male mice.

The differences between these studies may be due to the age differences. While our prior work evaluated locomotor behavior in male *Synj1*^+/−^ and *Synj1*^+/+^ mice at 7 and 12 months of age, in the current study we only evaluated male and female *Synj1*^+/−^ and *Synj1*^+/+^ mice starting at 7–8 months of age, with the purpose of conducting a broad characterization of motivation in *Synj1* mice, and early PD progression. Accordingly, most of the differences in our prior work were observed at 12 months of age, which is consistent with the fact that motor impairments in patients with *SYNJ1* mutations typically occur later in life (Tysnes and Storstein, 2017; Liou et al., 2020). However, we did previously observe moderate hyperactivity in mice at 7 months of age (Pan et al., 2020), which we did not observe in our present study owing to variability. Nonetheless, age-related deterioration in our previous study was more substantial at 12 months of age, with both male *Synj1*^+/−^ and *Synj1*^+/+^ mice showing deterioration in their activity levels. Additionally, *Synj1*^+/−^ mice showed specific signs of impaired motor coordination (Pan et al., 2020). Thus, given our observations of altered DA kinetics, we would predict that an age-related decline may occur more quickly in *Synj1*^+/−^ mice. Alternatively, deficits in gross motor activity may present themselves more obviously in tasks that require greater coordination—when motor systems and dopamine functions are pushed to their limits—such as the beam walking assay or rotarod.

### 4.3 Sex differences in DA-related psychiatric disorders

There are well-known sex differences in dopamine signaling. For example, in post-mortem human brains without documented psychiatric disorders, females have been found to have higher rates of DA turnover than males in the striatum (Konradi et al., 1992). Additionally, in psychiatric disorders where abnormal synaptic vesicular function is seen, females seem to be affected to a lesser degree than males (Carvalho-Netto et al., 2011; Kwon and Chapman, 2011). The resilience conferred in females is likely to account for sex differences that have been previously observed in schizophrenia, substance use disorders and PD (Carvalho-Netto et al., 2011; Kwon and Chapman, 2011; Calipari et al., 2017; Rajsombath et al., 2019).

In PD specifically, the accumulation of clathrin coated vesicles at nerve terminals has been observed alongside dystrophic changes in dopaminergic axon terminals, where men have a two times higher chance of developing the disease than women (Cremona et al., 1999; Chang-Ileto et al., 2011; Cerri et al., 2019). Additionally, in mouse models of PD, female hormones such as estradiol have been shown to significantly reduce parkinsonian-like symptoms and increase dopaminergic nerve fibers in males (Rajsombath et al., 2019). Our data demonstrates that *Synj1* deficiencies result in abnormal mesolimbic DA signaling in both males and females, however our data that illustrates that females metabolize cocaine at a slower rate, may be related to neurosteroid differences that are highly consistent with published findings where female mice do not exhibit parkinsonian-like symptoms to the same extent that male mice do. These sex differences indicate that mutations of the *Synj1* gene may be more detrimental to males than females, where differential treatment outcomes may be observed in males in relation to dopamine agonists (Rajsombath et al., 2019).

### 4.4 Implications for treatments

Our findings demonstrate that *Synj1* deficiency leads to differential expression of DAT—a major contributor of abnormal DA signaling in DA-related psychiatric disorders. Moreover, we report here that *Synj1* deficient mice exhibit delayed DA kinetics and slower peak in NAc DA levels in response to experimenter-administered cocaine. These findings bear important implications for Synj1 as a therapeutic target for DA-associated neurological and neuropsychiatric disorders, rather than DA replacement therapies which provide diminishing therapeutic benefits over long-term use in certain individuals (Marsden, 1994; Masoud et al., 2015). Overall, our data indicates that novel therapeutics capable of restoring deficits in vesicular trafficking and synaptic function, like *SYNJ1*, may be a new avenue that needs to be pursued to help treat individuals who may suffer from DA-associate neurological and neuropsychiatric disorders.

## Data availability statement

The raw data supporting the conclusions of this article will be made available by the authors, without undue reservation.

## Ethics statement

The animal study was approved by Rutgers University Institutional Animal Care and Use Committee. The study was conducted in accordance with the local legislation and institutional requirements.

## Author contributions

JM: Investigation, Data Curation, Writing – original draft, Writing – review & editing. JS: Investigation, Data Curation, Writing – original draft, Writing – review & editing. CO’B: Investigation, Data Curation, Writing – original draft, Writing – review & editing. CP: Investigation, Data Curation, Writing – original draft, Writing – review & editing. P-YP: Conceptualization, Data curation, Funding acquisition, Investigation, Methodology, Project administration, Resources, Supervision, Writing – original draft, Writing – review & editing. DB: Conceptualization, Data curation, Funding acquisition, Investigation, Methodology, Project administration, Resources, Supervision, Writing – original draft, Writing – review & editing.
